# End-tidal Carbon Dioxide Trajectory-based Prognostication of Out-of-hospital Cardiac Arrest

**DOI:** 10.5811/westjem.18403

**Published:** 2024-06-11

**Authors:** Chih-Hung Wang, Tsung-Chien Lu, Joyce Tay, Cheng-Yi Wu, Meng-Che Wu, Chun-Yen Huang, Chu-Lin Tsai, Chien-Hua Huang, Matthew Huei-Ming Ma, Wen-Jone Chen

**Affiliations:** * National Taiwan University, College of Medicine, Department of Emergency Medicine, Taipei, Taiwan; † National Taiwan University Hospital, Department of Emergency Medicine, Taipei, Taiwan; ‡ Far Eastern Memorial Hospital, Department of Emergency Medicine, New Taipei City, Taiwan; § National Taiwan University Hospital Yunlin Branch, Department of Emergency Medicine, Yunlin County, Taiwan

**Keywords:** Cardiopulmonary resuscitation, end-tidal carbon dioxide, group-based trajectory modeling, out-of-hospital cardiac arrest, survival, trajectory

## Abstract

**Background:**

During cardiopulmonary resuscitation (CPR), end-tidal carbon dioxide (EtCO_2_) is primarily determined by pulmonary blood flow, thereby reflecting the blood flow generated by CPR. We aimed to develop an EtCO_2_ trajectory-based prediction model for prognostication at specific time points during CPR in patients with out-of-hospital cardiac arrest (OHCA).

**Methods:**

We screened patients receiving CPR between 2015–2021 from a prospectively collected database of a tertiary-care medical center. The primary outcome was survival to hospital discharge. We used group-based trajectory modeling to identify the EtCO_2_ trajectories. Multivariable logistic regression analysis was used for model development and internally validated using bootstrapping. We assessed performance of the model using the area under the receiver operating characteristic curve (AUC).

**Results:**

The primary analysis included 542 patients with a median age of 68.0 years. Three distinct EtCO_2_ trajectories were identified in patients resuscitated for 20 minutes (min): low (average EtCO_2_ 10.0 millimeters of mercury [mm Hg]; intermediate (average EtCO_2_ 26.5 mm Hg); and high (average EtCO_2_: 51.5 mm Hg). Twenty-min EtCO_2_ trajectory was fitted as an ordinal variable (low, intermediate, and high) and positively associated with survival (odds ratio 2.25, 95% confidence interval [CI] 1.07–4.74). When the 20-min EtCO_2_ trajectory was combined with other variables, including arrest location and arrest rhythms, the AUC of the 20-min prediction model for survival was 0.89 (95% CI 0.86–0.92). All predictors in the 20-min model remained statistically significant after bootstrapping.

**Conclusion:**

Time-specific EtCO_2_ trajectory was a significant predictor of OHCA outcomes, which could be combined with other baseline variables for intra-arrest prognostication. For this purpose, the 20-min survival model achieved excellent discriminative performance in predicting survival to hospital discharge.

Population Health Research Capsule What do we already know about this issue?

*The end-tidal carbon dioxide (EtCO_2_) level during cardiopulmonary resuscitation (CPR) is associated with outcomes following out-of-hospital cardiac arrest (OHCA).*

What was the research question?

*Could EtCO_2_ trajectories during CPR be combined with baseline variables to predict outcomes of OHCA?*

What was the major finding of the study?

*The area under the curve of the EtCO_2_-based model for survival was 0.89 (95% confidence interval 0.86–0.92).*

How does this improve population health?

*An EtCO_2_ trajectory-based prediction model may help emergency medical services to predict OHCA outcomes and facilitate allocation of medical resources.*

## INTRODUCTION

The annual incidence of out-of-hospital cardiac arrest (OHCA) is estimated to be 28–44 cases per 100,000 population worldwide.^1^ The estimated proportion of survival to discharge in OHCA was 7.6% in Europe, 6.8% in North America, 3.0% in Asia, and 9.7% in Australia.^1^ High-quality cardiopulmonary resuscitation (CPR) is critical in improving OHCA outcomes.^2^^,^^3^ Capnography is recommended to monitor CPR quality in real time and adjust chest compression quality accordingly.^2^^,^^3^ During CPR, end-tidal carbon dioxide (EtCO_2_) is primarily determined by pulmonary blood flow, thereby reflecting the blood flow generated by CPR.^4^^,^^5^

The 2020 International Liaison Committee on Resuscitation (ILCOR) consensus^6^^,^^7^ recommended that EtCO_2_ ≥20 millimeters of mercury (mm Hg) measured after 20 minutes (min) of CPR may predict survival to discharge. Nonetheless, this weak recommendation was supported by only moderate-quality evidence. A 2018 ILCOR systematic review noticed that the measurement time points of EtCO_2_ were very heterogeneous across different studies.^8^ Accordingly, ILCOR^6^^,^^7^ suggested that instead of single EtCO_2_ values, the EtCO_2_ trend should be further explored in future studies for its prognostic performance.

The previous study noted that EtCO_2_ trajectory during CPR was associated with OHCA outcomes.^9^ However, the predictive ability of EtCO_2_ trajectory at a specific timing was not explored in the previous study.^9^ Whether EtCO_2_ can be combined with other metrics for intra-arrest prognostication was considered a critical knowledge gap by the 2020 American Heart Association (AHA) guidelines.^2^ In our recent study,^10^ we incorporated the minimum EtCO_2_ value into the return of spontaneous circulation after cardiac arrest (RACA) score and improved the performance of RACA score in predicting ROSC, suggesting that EtCO_2_ could potentially help intra-arrest prognostication.

In the current study, we further developed models that could predict survival at hospital discharge. Instead of a single EtCO_2_ value,^10^ we attempted to combine EtCO_2_ trajectory and other predictors in deriving prediction models. Moreover, these models were developed using time-specific windows to prognosticate patient outcomes during resuscitation, including 10- and 20 min^6^^,^^7^ after initiation of CPR.

## MATERIALS AND METHODS

This observational study was a secondary analysis of a prospectively collected OHCA database registered in the emergency department (ED) of National Taiwan University Hospital (NTUH). The institutional review board approved this study (reference number: 201906082RINB) and waived the requirement for informed consent. The study was performed according to the recommendations from Worster et al^11^ regarding health record review studies in emergency medicine research with all elements followed. The results are reported according to the transparent reporting of a multivariable prediction model for individual prognosis or diagnosis (TRIPOD) statement.^12^

### Study Setting

The NTUH is a tertiary-care medical center with 2,600 beds, including 220 beds in intensive care units. Approximately 100,000 patients visit NTUH ED annually. Patients with OHCA are transported directly to the resuscitation bay of the critical care area in the ED for CPR, which is delivered according to resuscitation guidelines.^2^^,^^3^ Also, since 2013 ED staff have been trained with the A-C-L-S (airway-circulation-leadership-support) teamwork model^9^^,^^13^^,^^14^ to streamline the resuscitation process via both strengthened technical and non-technical skills.^15^^,^^16^ Any intervention, such as tracheal intubation performed during CPR, are timestamped by nurses with a specially designed mobile application. The EtCO_2_ is recorded every two min right before pulse check. The EtCO_2_ is monitored with devices attached to the advanced airways, including supraglottic airways and endotracheal tubes. For patients with OHCA who never achieve return of spontaneous circulation (ROSC), CPR is usually performed for at least 30 min in the ED, except for those with a documented do-not-resuscitate (DNR) order.

### Study Population

Patients with OHCA sent to the NTUH ED between January 1, 2015–December 31, 2021 were screened. The inclusion criteria for the study were as follows: 1) non-traumatic arrest; 2) absence of ROSC before ED arrival; (3) absence of documented DNR order before CPR; 4) age ≥18 years; and 5) insertion of advanced airways during CPR. Based on the CPR duration, the included patients would be further selected for primary and secondary analyses. If the included patients received CPR ≥20 min and had EtCO_2_ measurements ≥3 times within 20 min of CPR, they would be selected into the 20-min group for the primary analysis. Similarly, if the included patients received CPR ≥10 min and had EtCO_2_ measurements ≥3 times within 10 min of CPR, they would be selected into the 10-min group for secondary analysis.

### Data Collection, Variable Definitions, and Outcome Measures

In the NTUH database, OHCA events were recorded based on the Utstein template.^17^ Data requested for analysis included age, gender, variables derived from the Utstein template, advanced airway insertion timing, EtCO_2_ values with measurement timing, and outcomes. For ED resuscitation, the time point of the initial chest compression delivered in the ED was set as time zero for reference. Time to advanced airway use was defined as the interval between time zero and time for completing advanced airway insertion. If advanced airway devices were inserted before ED arrival, the time to advanced airway was recorded as zero. Duration of CPR in the ED referred to the time interval between time zero and the end of resuscitation, either due to ROSC or death. Time-specific EtCO_2_ referred to the EtCO_2_ level measured after the specific time elapsing following time zero.

The primary outcome was survival status at the time of hospital discharge. The secondary outcome was ROSC, defined as a palpable pulse for 20 seconds.^18^ Data abstraction for the current analysis was performed by trained researchers who were blinded to the study hypothesis.

### Statistical Analysis

In the primary analysis, we used the 20-min group to build models for predicting survival (20-min survival model) and ROSC (20-min ROSC model). In the secondary analysis, similar procedures were applied to develop the 10-min survival model and 10-min ROSC model. We first performed group-based trajectory modeling (GBTM) to identify trajectory groups based on the EtCO_2_ level. The GBTM is an explanatory modeling technique to identify hidden groups of individuals with similar trajectories for a particular variable of interest.^19^ The GBTM performs better when longitudinal data is measured at least three times.

For descriptive statistics, categorical variables are presented as proportions, and continuous variables are presented as medians with interquartile ranges. We examined categorical variables using the chi-squared test, whereas continuous variables were compared using the Kruskal-Wallis test or Mann-Whitney test, as appropriate. We used multivariable logistic regression analyses to develop the prediction models. All available variables, including basic demographics, peri-CPR events, and EtCO_2_ trajectory were accounted for in the regression model via a stepwise, variable selection procedure. The EtCO_2_ trajectory would be tested as ordinal or categorical variables in the model-building process. We used generalized additive models (GAM)^20^ to identify the appropriate cutoff point(s) for dichotomization. The discriminative performance and calibration of the prediction model were assessed by area under the receiver operating characteristic curve (AUC) and the Hosmer-Lemeshow goodness-of-fit test, respectively. We internally validated the prediction model using the bootstrapping procedure with 1,000 repetitions to examine the robustness of the effect estimate of each variable in the prediction model.

We performed GBTM and bootstrapping using the traj package and bootstrap procedure of Stata software (StataCorp LLC, College Station, TX), respectively. We used the R 4.1.1 software (R Foundation for Statistical Computing, Vienna, Austria) for other analyses. A two-tailed *P*-value <0.05 was considered statistically significant.

## RESULTS

**Figure 1.**
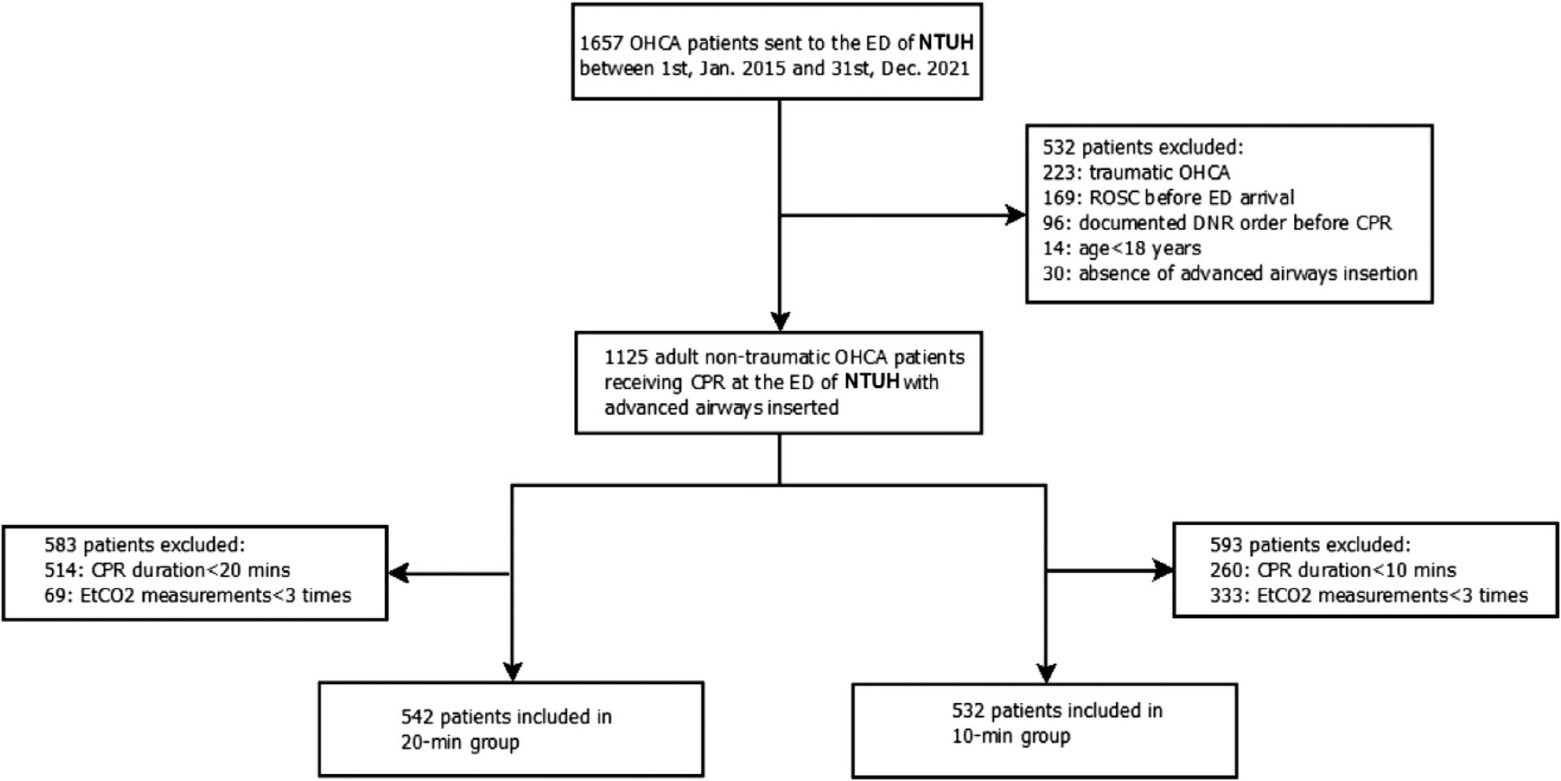
Patient inclusion flowchart. *CPR*, cardiopulmonary resuscitation; *DNR*, do-not-resuscitate; *ED*, emergency department; *NTUH*, National Taiwan University Hospital; *OHCA*, out-of-hospital cardiac arrest; *ROSC*, return of spontaneous circulation.

The patient selection procedure resulted in 542 and 532 patients in the 20-min and 10-min groups, respectively (Figure 1). The two groups were not mutually exclusive. Because not all patients in the 20-min group had EtCO_2_ measurements ≥3 times within 10 mins, the 20-min group patients may not have been necessarily included in the 10-min group. Also, because some of the patients in the 10-min group would achieve ROSC within 20 min of CPR, the 10-min group patients would not necessarily have been included in the 20-min group. Therefore, there was an overlap of 385 patients between the 20-min and 10-min groups who met the selection criteria for both groups.

**Figure 2.**
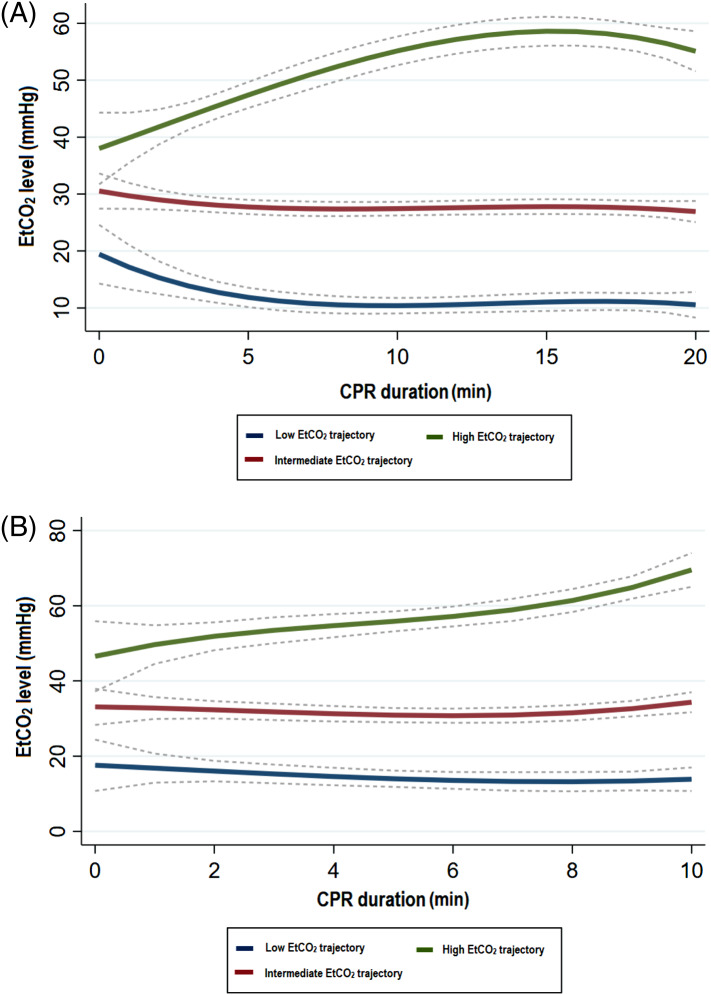
The end-tidal carbon dioxide trajectory. The EtCO_2_ trajectory groups identified by group-based trajectory modeling in the (A) primary (20 minute) and (B) secondary (10 minute) analysis. Dotted lines indicate 95% confidence intervals. *CPR*, cardiopulmonary resuscitation; *EtCO*_*2*_, end-tidal carbon dioxide.

In the primary analysis, we identified and named three EtCO_2_ trajectories as low, intermediate, and high trajectories according to their respective average EtCO_2_ levels (Figure 2). The characteristics of the 20-min group and comparisons between these EtCO_2_ trajectories are presented in Table 1. The median CPR duration in the ED was 31.0 minutes, and the median number of EtCO_2_ measurements was eight. A total of 25 (4.6%) patients survived at hospital discharge. There seems to be an increasing trend of survival from low to high EtCO_2_ trajectory. The comparisons between patients stratified by survival are shown in Supplemental Table 1. During the model development, the 20-min EtCO_2_ trajectory was fitted as an ordinal variable by the logistic regression analysis and positively associated with survival (odds ratio [OR] 2.25, 95% confidence interval [CI] 1.07–4.74) and ROSC (OR 2.46, 95% CI 1.78–3.41) (Table 2). In other words, compared with the low EtCO_2_ trajectory, the intermediate trajectory had 2.25 times higher odds of survival to hospital discharge. Similarly, compared with the intermediate trajectory, the high EtCO_2_ trajectory also had 2.25 times higher odds of survival. When the 20-min EtCO_2_ trajectory was combined with other variables, the AUCs of the 20-min survival and ROSC models were 0.89 (95% CI 0.86–0.92) and 0.78 (95% CI 0.74–0.81), respectively.

**Table 1.** Characteristics of patients included in the twenty-minute group stratified by end-tidal carbon dioxide trajectory group.

Variables	Twenty-min group (n = 542)	Twenty-min low EtCO_2_ trajectory (n = 196)	Twenty-min intermediate EtCO_2_ trajectory (n = 280)	Twenty-min high EtCO_2_ trajectory (n = 66)	*P* value
Basic demographics					
Age, year	68.0 (57.0–80.0)	70.0 (58.0–80.0)	67.0 (56.0–81.0)	66.0 (56.0–76.0)	0.45
Male, n	354 (65.3)	111 (56.6)	199 (71.1)	44 (66.7)	0.005
Peri-CPR events					
Transported by EMS, n	507 (93.5)	179 (91.3)	263 (93.9)	65 (98.5)	0.11
Arrest at home, n	296 (54.6)	113 (57.6)	149 (53.2)	34 (51.5)	0.55
Witness by bystander, n	193 (35.6)	56 (28.5)	112 (40.0)	25 (37.9)	0.03
Witness by EMS, n	28 (5.2)	11 (5.6)	12 (4.3)	5 (7.6)	0.52
Witness by bystander or EMS, n	212 (39.1)	61 (31.1)	121 (43.2)	30 (45.4)	0.02
Bystander CPR, n	269 (49.6)	93 (47.4)	140 (50.0)	36 (54.5)	0.60
Prehospital defibrillation by EMS, n	117 (21.5)	17 (8.6)	82 (29.2)	18 (27.2)	<0.001
Initial shockable rhythms at ED arrival, n	37 (6.8)	8 (4.1)	25 (8.9)	4 (6.1)	0.43
Duration of prehospital CPR performed by EMS, min	17.0 (12.0–21.0)	17.0 (10.5–21.0)	17.0 (12.0–21.0)	18.0 (12.0–22.0)	0.30
Procedures during CPR					
SGA use, n	376 (69.4)	134 (68.4)	196 (70.0)	46 (69.7)	0.93
Time to SGA use, min	0 (0–0) (n = 376)	0 (0–0) (n = 134)	0 (0–0) (n = 196)	0 (0–0) (n = 46)	0.12
ETT use, n	531 (98.0)	189 (96.4)	277 (98.9)	65 (98.5)	0.12
Time to ETT use, min	3.0 (2.0–5.0) (n = 531)	3.0 (2.0–6.0) (n = 189)	3.0 (2.0–5.0) (n = 277)	3.0 (1.5–4.0) (n = 65)	0.30
Time-specific EtCO_2_ levels, mmHg					
0-min EtCO_2_	29.0 (20.3–36.0) (n = 39)	15.0 (12.5–20.5) (n = 8)	32.0 (25.3–36.0) (n = 27)	32.0 (25.0–50.5) (n = 4)	0.003
1-min EtCO_2_	24.5 (15.0–38.5) (n = 56)	14.5 (10.5–19.0) (n = 20)	28.0 (22.0–38.3) (n = 25)	36.3 (24.0–68.3) (n = 11)	<0.001
2-min EtCO_2_	24.0 (5.8–33.0) (n = 113)	14.0 (9.0–23.3) (n = 37)	27.5 (20.0–33.0) (n = 62)	41.0 (24.0–54.0) (n = 14)	<0.001
3-min EtCO_2_	22.0 (13.5–36.0) (n = 120)	11.5 (6.0–20.0) (n = 46)	30.0 (21.0–39.5) (n = 60)	36.5 (23.0–43.0) (n = 14)	<0.001
4-min EtCO_2_	22.0 (12.0–33.0) (n = 231)	11.5 (7.0–18.0) (n = 78)	24.0 (18.0–34.8) (n = 123)	44.0 (30.0–52.0) (n = 30)	<0.001
5-min EtCO_2_	22.0 (12.0–33.0) (n = 121)	10.0 (3.0–14.8) (n = 43)	27.0 (21.0–35.0) (n = 62)	41.0 (30.5–60.0) (n = 16)	<0.001
6-min EtCO_2_	21.0 (12.0–31.0) (n = 245)	8.0 (3.0–12.0) (n = 75)	24.0 (18.0–31.0) (n = 141)	47.0 (37.8–60.8) (n = 29)	<0.001
7-min EtCO_2_	18.5 (10.0–32.0) (n = 142)	9.0 (4.0–13.5) (n = 61)	27.0 (18.0–34.0) (n = 62)	44.0 (30.0–62.3) (n = 19)	<0.001
8-min EtCO_2_	22.0 (11.0–34.0) (n = 282)	9.0 (3.0–12.0) (n = 94)	27.0 (19.0–35.3) (n = 157)	56.0 (45.0–60.8) (n = 31)	<0.001
9-min EtCO_2_	20.0 (10.0–34.0) (n = 147)	8.5 (3.0–12.0) (n = 58)	27.0 (19.0–36.0) (n = 70)	58.0 (45.0–72.8) (n = 19)	<0.001
10-min EtCO_2_	21.0 (12.0–33.0) (n = 296)	9.0 (3.3–13.0) (n = 103)	27.0 (20.0–34.8) (n = 163)	52.5 (48.0–68.0) (n = 30)	<0.001
11-min EtCO_2_	21.0 (11.0–36.5) (n = 144)	11.0 (5.0–15.0) (n = 58)	28.0 (21.0–36.0) (n = 63)	60.0 (45.0–65.8) (n = 23)	<0.001
12-min EtCO_2_	21.0 (12.0–31.8) (n = 331)	10.0 (5.0–14.0) (n = 122)	26.0 (21.0–33.0) (n = 176)	58.0 (43.8–71.3) (n = 33)	<0.001
13-min EtCO_2_	21.0 (12.0–33.8) (n = 123)	9.5 (7.5–13.5) (n = 48)	26.0 (20.8–33.0) (n = 57)	51.5 (44.0–65.0) (n = 18)	<0.001
14-min EtCO_2_	21.0 (12.0–33.0) (n = 324)	10.0 (5.0–15.0) (n = 117)	26.0 (21.0–34.0) (n = 173)	53.0 (45.0–69.0) (n = 34)	<0.001
15-min EtCO_2_	21.0 (11.0–32.0) (n = 143)	9.5 (4.0–14.0) (n = 58)	27.0 (21.0–35.0) (n = 65)	50.0 (43.0–58.5) (n = 20)	<0.001
16-min EtCO_2_	22.0 (12.0–33.0) (n = 329)	9.0 (6.0–14.0) (n = 114)	26.5 (21.0–33.0) (n = 180)	59.0 (47.3–68.8) (n = 35)	<0.001
17-min EtCO_2wp_	21.0 (12.0–36.0) (n = 139)	9.0 (5.0–13.5) (n = 52)	27.0 (21.0–33.8) (n = 63)	56.0 (45.5–66.5) (n = 24)	<0.001
18-min EtCO_2_	21.0 (10.8–32.0) (n = 333)	9.0 (3.0–14.0) (n = 125)	26.0 (20.0–33.0) (n = 173)	55.0 (43.0–69.0) (n = 35)	<0.001
19-min EtCO_2_	21.0 (10.0–34.0) (n = 137)	8.5 (3.0–13.0) (n = 50)	23.0 (20.0–34.0) (n = 68)	50.0 (44.3–62.0) (n = 19)	<0.001
20-min EtCO_2_	21.0 (11.0–33.3) (n = 329)	9.0 (4.5–14.0) (n = 123)	26.0 (20.0–34.0) (n = 171)	56.0 (50.0–64.5) (n = 35)	<0.001
Available measurements of EtCO_2_ levels, times	8.0 (6.0–9.0)	8.0 (7.0–9.0)	8.0 (6.0–9.0)	8.0 (7.0–9.0)	0.64
EtCO_2_ summary parameters, mm Hg					
Initial	23.0 (14.0–36.0)	14.0 (7.0–20.5)	29.0 (20.0–41.0)	41.5 (28.0–61.0)	<0.001
Maximum	36.0 (22.0–50.0)	18.0 (12.0–24.0)	41.0 (34.0–49.0)	69.0 (63.0–79.0)	<0.001
Minimum	13.0 (5.0–21.0)	3.5 (2.0–9.0)	16.0 (12.0–22.0)	30.0 (23.0–41.0)	<0.001
Final	21.0 (11.0–35.0)	9.0 (4.0–14.0)	26.0 (20.0–34.5)	56.0 (46.0–65.0)	<0.001
Average	23.0 (14.0–33.0)	10.0 (6.5–14.0)	26.5 (22.0–33.0)	51.5 (37.0–58.0)	<0.001
Duration of CPR performed in ED, min	31.0 (30.0–35.0)	31.0 (30.0–34.0)	31.0 (30.0–36.0)	31.0 (30.0–33.0)	0.50
Outcome, n					
ROSC	184 (33.9)	32 (16.3)	118 (42.1)	34 (51.5)	<0.001
Survival to hospital discharge	25 (4.6)	3 (1.5)	16 (5.7)	6 (9.1)	0.02

Data are presented as median (interquartile range) or counts (proportion).

*CPR*, cardiopulmonary resuscitation; *ED*, emergency department; *EMS*, emergency medical services; *mm Hg*, millimeters of mercury; *ETT*, endotracheal tube; *ROSC*, return of spontaneous circulation; *SGA*, supraglottic airway; min, minute.

**Table 2.** Multivariable logistic regression analysis for twenty-minute group to build end-tidal carbon dioxide trajectory-based prediction models.

Variables	Odds ratio (95% confidence interval)	*P* value
*Twenty-min survival model*		
Twenty-min EtCO_2_ trajectory	2.25 (1.07–4.74)	0.03
Arrest at home	0.28 (0.10–0.77)	0.01
Prehospital defibrillation by EMS	3.42 (1.34–8.77)	0.01
Initial shockable rhythms at ED arrival	8.36 (3.13–22.31)	<0.001
*Twenty-min ROSC model*		
Twenty-min EtCO_2_ trajectory	2.46 (1.78–3.41)	<0.001
Arrest at home	0.54 (0.34–0.85)	0.008
Witness by bystander or EMS	1.72 (1.13–2.63)	0.01
Prehospital defibrillation by EMS	2.72 (1.64–4.53)	<0.001
Initial shockable rhythms at ED arrival	4.97 (2.07–11.90)	<0.001
Duration of prehospital CPR performed by EMS	0.96 (0.93–0.99)	0.003

Twenty-min survival model: goodness-of-fit assessment: n = 542, adjusted generalized *R* ^2^ = 0.32, estimated area under the receiver operating characteristic curve = 0.89 (95% confidence interval: 0.86–0.92), and Hosmer and Lemeshow goodness-of-fit Chi-Squared test *p* = 0.64; Twenty-min ROSC model: goodness-of-fit assessment: n = 542, adjusted generalized *R* ^2^ = 0.30, estimated area under the receiver operating characteristic curve = 0.78 (95% confidence interval: 0.74–0.81), and Hosmer and Lemeshow goodness-of-fit Chi-Squared test *p* = 0.19.

*CPR*, cardiopulmonary resuscitation; *ED*, emergency department; *EMS*, emergency medical services; *ROSC*, return of spontaneous circulation; *min*, minute.

Similarly, in the secondary analysis we identified three EtCO_2_ trajectories (Figure 2 and Table 3). The median CPR duration in the ED was 30.0 min, and the median number of EtCO_2_ measurements was four. A total of 34 (6.4%) patients survived at hospital discharge. Significant survival differences were noted among the three EtCO_2_ trajectories; nonetheless, the survival of intermediate and high EtCO_2_ trajectories was similar. The survival-stratified comparisons are shown in Supplemental Table 2. During the model-fitting process, the 10-min EtCO_2_ trajectory was fitted as a categorical variable. As shown in Table 4, compared with the 10-min low EtCO_2_ trajectory, the 10-min intermediate or high EtCO_2_ trajectory was significantly associated with survival (OR 2.53, 95% CI 1.10–5.81). In addition, compared with the 10-min low EtCO_2_ trajectory, 10-min intermediate (OR 3.36, 95% CI 2.25–5.04) and high (OR 6.59, 95% CI 3.42–12.69) EtCO_2_ trajectories were significantly associated with ROSC, respectively. When the 10-min EtCO_2_ trajectory was combined with other variables, the AUC of the 10-min survival and ROSC models were 0.76 (95% CI 0.72–0.79) and 0.75 (95% CI 0.71–0.79), respectively.

**Table 3.** Characteristics of patients included in the ten-min group stratified by end-tidal carbon dioxide trajectory group.

Variables	Ten-min group (n = 532)	Ten-min low EtCO_2_ trajectory (n = 234)	Ten-min intermediate EtCO_2_ trajectory (n = 240)	Ten-min high EtCO_2_ trajectory (n = 58)	*P* value
Basic demographics					
Age, year	71.0 (59.5–82.0)	73.0 (60.0–84.0)	70.0 (60.0–81.0)	70.5 (56.0–79.0)	0.22
Male, n	346 (65.0)	143 (61.1)	167 (69.6)	36 (62.1)	0.14
Peri-CPR events					
Transported by EMS, n	500 (94.0)	215 (91.9)	227 (94.6)	58 (100)	0.11
Arrest at home, n	308 (57.9)	144 (61.5)	134 (55.8)	34 (51.7)	0.27
Witness by bystander, n	192 (36.1)	78 (33.3)	89 (37.1)	25 (43.1)	0.35
Witness by EMS, n	26 (4.9)	7 (3.0)	16 (6.7)	3 (5.2)	0.18
Witness by bystander or EMS, n	207 (38.9)	79 (33.8)	101 (42.1)	27 (46.6)	0.08
Bystander CPR, n	276 (51.9)	115 (49.1)	126 (52.5)	35 (60.3)	0.30
Prehospital defibrillation by EMS, n	101 (19.0)	24 (10.2)	60 (25.0)	17 (29.3)	<0.001
Initial shockable rhythms at ED arrival, n	30 (5.6)	11 (4.7)	16 (6.7)	3 (5.2)	0.64
Duration of prehospital CPR performed by EMS, min	17.0 (12.0–21.0)	18.0 (11.0–21.0)	17.0 (12.0–21.0)	18.0 (14.0–22.0)	0.44
Procedures during CPR					
SGA use, n	380 (71.4)	166 (70.9)	172 (71.7)	42 (72.4)	0.97
Time to SGA use, min	0 (0–0) (n = 380)	0 (0–0) (n = 166)	0 (0–0) (n = 172)	0 (0–0) (n = 42)	0.24
ETT use, n	508 (95.5)	219 (93.6)	234 (97.5)	55 (94.8)	0.12
Time to ETT use, min	3.0 (2.0–4.0) (n = 508)	3.0 (2.0–4.0) (n = 219)	3.0 (2.0–4.0) (n = 234)	3.0 (1.3–4.0) (n = 55)	0.48
Time-specific EtCO_2_ levels, mm Hg					
0-min EtCO_2_	26.0 (18.0–36.0) (n = 48)	18.0 (14.5–20.5) (n = 16)	31.0 (25.3–38.3) (n = 27)	55.0 (28.5–60.8) (n = 5)	<0.001
1-min EtCO_2_	24.0 (12.0–38.3) (n = 73)	12.0 (7.0–17.0) (n = 30)	32.5 (22.0–41.0) (n = 34)	56.0 (40.5–69.3) (n = 9)	<0.001
2-min EtCO_2_	25.5 (17.0–37.5) (n = 148)	17.0 (11.0–23.0) (n = 62)	32.5 (23.0–42.0) (n = 70)	54.5 (45.0–61.5) (n = 16)	<0.001
3-min EtCO_2_	24.0 (14.0–36.0) (n = 158)	13.5 (9.0–21.0) (n = 70)	34.0 (25.3–43.8) (n = 71)	48.0 (22.8–54.0) (n = 17)	<0.001
4-min EtCO_2_	23.0 (13.3–35.8) (n = 299)	13.0 (9.0–19.8) (n = 131)	30.0 (22.0–38.0) (n = 132)	51.0 (45.5–62.5) (n = 36)	<0.001
5-min EtCO_2_	23.0 (12.0–34.0) (n = 153)	12.0 (3.5–17.0) (n = 63)	29.0 (23.0–36.0) (n = 74)	60.0 (42.0–66.5) (n = 16)	<0.001
6-min EtCO_2_	22.0 (13.0–34.0) (n = 326)	12.0 (7.0–18.0) (n = 142)	28.0 (21.3–38.0) (n = 147)	56.0 (42.8–63.3) (n = 37)	<0.001
7-min EtCO_2_	23.0 (10.0–36.0) (n = 154)	10.0 (5.5–15.5) (n = 68)	30.0 (24.8–37.0) (n = 69)	55.0 (45.8–64.8) (n = 17)	<0.001
8-min EtCO_2_	25.0 (13.0–38.0) (n = 343)	12.0 (7.8–17.3) (n = 149)	33.0 (26.0–40.0) (n = 159)	62.0 (56.3–72.5) (n = 35)	<0.001
9-min EtCO_2_	23.0 (11.0–37.0) (n = 142)	10.0 (4.0–15.8) (n = 63)	30.0 (23.0–37.0) (n = 60)	62.0 (54.5–78.0) (n = 19)	<0.001
10-min EtCO_2_	23.0 (14.0–39.8) (n = 339)	13.0 (6.0–18.0) (n = 150)	32.0 (24.0–43.0) (n = 154)	68.0 (58.0–79.5) (n = 35)	<0.001
Available measurements of EtCO_2_ levels, times	4.0 (3.0–5.0)	4.0 (3.0–5.0)	4.0 (3.0–5.0)	4.0 (4.0–5.0)	0.41
EtCO_2_ summary parameters, mm Hg					
Initial	25.0 (15.0–40.0)	15.0 (10.0–22.0)	34.0 (25.0–43.5)	55.5 (45.0–65.0)	<0.001
Maximum	34.0 (22.0–50.0)	20.0 (13.0–26.0)	44.0 (36.0–51.0)	71.5 (63.0–89.0)	<0.001
Minimum	16.0 (9.0–24.5)	8.0 (3.0–12.0)	21.5 (17.0–27.0)	41.5 (33.0–54.0)	<0.001
Final	23.0 (13.0–39.0)	12.0 (6.0–18.0)	33.0 (24.0–42.5)	64.0 (57.0–78.0)	<0.001
Average	25.0 (15.0–36.0)	13.0 (8.0–19.0)	32.0 (26.0–37.0)	58.0 (51.0–64.0)	<0.001
Duration of CPR performed in ED, min	30.0 (18.0–32.0)	30.0 (22.0–32.0)	30.0 (17.0–33.0)	20.0 (13.0–31.0)	0.008
Outcome, n					
ROSC	239 (44.9)	64 (27.4)	135 (56.3)	40 (69.0)	<0.001
Survival to hospital discharge	34 (6.4)	8 (3.4)	21 (8.8)	5 (8.6)	0.05

Data are presented as median (interquartile range) or counts (proportion).

*CPR*, cardiopulmonary resuscitation; *ED*, emergency department; *EMS*, emergency medical service; *mm HG*, millimeters of mercury; *ETT*, endotracheal tube; *ROSC*, return of spontaneous circulation; *SGA*, supraglottic airway.

**Table 4.** Multivariable logistic regression analysis for ten-minute group to build end-tidal carbon dioxide trajectory-based prediction models.

Variables	Odds ratio (95% confidence interval)	*P* value
*Ten-min survival model*	
Ten-min intermediate or high EtCO_2_ trajectory	2.53 (1.10–5.81)	0.03
Witness by bystander	3.00 (1.42–6.33)	0.004
Initial shockable rhythms at ED arrival	5.21 (2.03–13.33)	<0.001
*Ten-min ROSC model*		
Ten-min intermediate EtCO_2_ trajectory	3.36 (2.25–5.04)	<0.001
Ten-min high EtCO_2_ trajectory	6.59 (3.42–12.69)	<0.001
Age between 37 and 69 (year)	1.49 (1.02–2.20)	0.04
Witness by bystander or EMS	1.92 (1.31–2.84)	0.001
Initial shockable rhythms at ED arrival	5.29 (2.04–13.71)	<0.001
Duration of prehospital CPR performed by EMS (min)	0.96 (0.93–0.98)	<0.001

Ten-min survival model: goodness-of-fit assessment: n = 532, adjusted generalized *R* ^2^ = 0.14, estimated area under the receiver operating characteristic curve = 0.76 (95% confidence interval: 0.72–0.79), and Hosmer and Lemeshow goodness-of-fit chi-squared test *P* = 0.79; ten-min ROSC model: goodness-of-fit assessment: n = 532, adjusted generalized *R* ^2^ = 0.25, estimated area under the receiver operating characteristic curve = 0.75 (95% confidence interval: 0.71–0.79), and Hosmer and Lemeshow goodness-of-fit chi-squared test *P* = 0.65.

*CPR*, cardiopulmonary resuscitation; *ED*, emergency department; *EMS*, emergency medical service; *ROSC*, return of spontaneous circulation.

For the 20- and 10-min models, all the predictors remained significantly associated with outcomes after the bootstrapping procedure, indicating the robustness of these models (Supplemental Table 3).

## DISCUSSION

### Main Findings

By using a prospectively collected database, we identified that the time-specific EtCO_2_ trajectory was a significant intra-arrest outcome predictor. Time-specific EtCO_2_ trajectory could be combined with other predictors to assist in intra-arrest prognostication at different time points during CPR. Among all the prediction models, the 20-min EtCO_2_ trajectory-based survival model achieved the highest discriminative performance (AUC 0.89).

### Comparison with Previous Studies

For outcome prediction in OHCA, most models were developed for patients who had already achieved ROSC.^21^ There were few, if any, models available for patients who were still undergoing CPR. For predicting ROSC before CPR was performed, the RACA score^18^ was one of the most well-validated models, demonstrating AUC ranging from 0.71 to 0.76.^22^^–^^24^ All the predictors included in the RACA score were baseline variables, such as arrest location and arrest rhythms, which did not consider the treatment effects of CPR. Nonetheless, it was possible that even though the RACA score-predicted ROSC probabilities were similar, the actual outcomes may differ because of different CPR qualities and durations delivered by rescuers. To make individualized intra-arrest prognostication, variables specific to the patient and resuscitation process, such as EtCO_2_, may be necessary,.

The 2018 ILCOR systematic review^8^ indicated that EtCO_2_ was associated with ROSC probability. Nonetheless, the optimal parameter of EtCO_2_ for prognostication is still debated.^8^ For example, despite its convenience in statistical analysis, average EtCO_2_ could not differentiate between different EtCO_2_ trajectories. Ascending and descending EtCO_2_ trajectories may have similar average EtCO_2_, but their prognoses may be very different.^25^^,^^26^ Moreover, the term “initial” EtCO_2_ may not accurately reflect the EtCO_2_ level during the early phase of CPR, as the endotracheal tube could potentially be introduced later during the resuscitation. It was reported that the specificity of EtCO_2_ in predicting ROSC would increase progressively from 50% at 0 min to 60%, 98%, and 100% at 10, 15, and 20 min, respectively.^27^ Therefore, for EtCO_2_ to be a valid predictor, the timing of prognostication should be specified, and its trend during CPR, instead of a single value, should be adopted.

### Interpretation of Current Analysis

The 2020 ILCOR consensus^6^^,^^7^ recommends that EtCO_2_ measured after 20 min of CPR may be a predictor of survival to discharge. Rosman et al^28^ indicated that when higher EtCO_2_ levels were reached beyond 20 min of CPR they may not lead to ROSC. Progressively worsening ischemia may cause refractoriness to CPR during the metabolic phase of cardiac arrest,^29^ and EtCO_2_ trajectories beyond 20 min may not be prognostic of outcomes. Therefore, CPR for 20 min was used to select the 20-min cohort and identify the 20-min EtCO_2_ trajectory. The advantage of employing GBTM was that it offered an efficient method to unravel the hidden trajectories that may not be readily recognizable from the baseline characteristics or initial EtCO_2_ values. The significantly different EtCO_2_ levels among EtCO_2_ trajectories indicated the success of GBTM in distinguishing these hidden clusters (Table 1). Also, in an unbiased manner, GBTM identifies the hidden EtCO_2_ trajectories only by examining the repeatedly measured EtCO_2_ without considering baseline variables or outcomes. Whether the identified trajectories were associated with outcomes should be further investigated. For example, compared with patients with low 20-min EtCO_2_ trajectory, those with intermediate or high 20-min EtCO_2_ trajectory had higher proportions of bystander-witnessed arrest (Table 1), which may also explain better outcomes in the latter.

In the 20-min survival model, the multivariable logistical regression analysis indicated that the 20-min EtCO_2_ trajectory was positively associated with survival, demonstrating the trend of a higher EtCO_2_ trajectory with increased survival. Studies revealed that for every 10 mm increase in chest compression depth, EtCO_2_ would increase by 1.4 mm Hg^30^ or 4.0%.^31^ Higher EtCO_2_ trajectory may suggest better CPR quality, which may explain the positive association between EtCO_2_ trajectory and chances of survival. In contrast, arrest etiology may also be a confounding factor in explaining the associations between favorable outcomes and intermediate or high EtCO_2_ trajectory. Studies have shown that patients with asphyxial arrest^32^ or suspected respiratory etiology^33^ may have higher EtCO_2_ levels than those with initial shockable rhythms ^32^ or suspected cardiac etiology,^33^ respectively. Nonetheless, in our cohort, patients of intermediate or high EtCO_2_ trajectory had higher proportions of prehospital defibrillation by emergency medical services (EMS) (Table 1). Therefore, instead of the arrest etiology, the CPR quality may account for the positive association between 20-min EtCO_2_ trajectory and survival.

Whether EtCO_2_, along with other factors, can be used for intra-arrest prognostication was listed by AHA guidelines^2^ as an important knowledge gap. In the 20-min survival model, besides EtCO_2_ trajectory, other baseline variables, including arrest at home, prehospital defibrillation by EMS, and initial shockable rhythms on ED arrival, were also selected as significant predictors. These baseline variables had been well-validated for their predictive performance in previous studies.^18^ The 20-min survival model achieved excellent discriminative performance and may first answer the question presented by the AHA.^2^ Moreover, we further tested whether the 20-min EtCO_2_ trajectory could facilitate predicting ROSC. However, the AUC of the 20-min ROSC model was 0.78, lower than that of the 20-min survival model. In our study, ROSC was defined as a palpable pulse for 20 seconds, as used by RACA score.^18^ The swift nature of this secondary outcome may render it difficult to be predicted, even though the 20-min ROSC model included more variables than the 20-min survival model.

Finally, we developed the 10-min prediction models to explore whether outcomes could be predicted at an earlier time point during CPR. Nevertheless, the AUCs of both 10-min models were respectively lower than their counterparts of 20-min models. As shown in Figure 2, the 10-min EtCO_2_ trajectory was slightly different from the 20-min EtCO_2_ trajectory in the trend pattern. For example, the high EtCO_2_ trajectory continued to rise within 10 min; it was only evident later in the 20-min window that the trajectory had plateaued. Taken together, these time-specific models varied over time in terms of trajectory shapes and model performance. Earlier trajectories may still be evolving with moderate model performance, while late trajectories may have improved model performance at the cost of more medical recourses consumed. Our data suggested that 20 min after CPR may be the earliest point in time with excellent model performance to predict distant, clinically important outcomes, such as survival to hospital discharge.

### Future Applications

For OHCA patients transported to the ED for continuous CPR, emergency clinicians are faced with the problem of balancing the probability of a favorable outcome with the utilization of current and future resources when making important decisions, such as termination of resuscitation or implementation of invasive extracorporeal CPR.^34^ Most of these advanced interventions are reserved for patients receiving CPR within a certain duration.^34^ Despite the fact that CPR duration is known to be inversely associated with favorable outcomes,^35^ it may not be the sole prognostic factor. Quality CPR may facilitate maintaining patients’ potential for favorable outcomes and lengthen the time window for advanced interventions to be implemented. Our prediction models demonstrated that time-specific EtCO_2_ trajectory, taking into account both the CPR duration and quality, could be a significant intra-arrest prognostic factor. In the future, time-specific EtCO_2_ may be transmitted instantaneously from EtCO_2_ monitors to mobile devices with the assistance of advanced information and communication technology. The predicted outcomes could be updated instantaneously minute by minute for each individual patient and may not be restricted to a certain time point during CPR, such as 20 min or 10 min, as used in our study.

## LIMITATIONS

First, while we had internally validated the prediction models by using the bootstrap method, further external validation in other datasets should be performed. Second, the analyzed EtCO_2_ dataset was derived from a prospectively collected database of a single ED with a specialized training model for CPR. Further studies are needed to investigate whether these models could be generalized to other EDs or prehospital resuscitation.

## CONCLUSION

Time-specific EtCO_2_ trajectory was a significant predictor of OHCA outcomes, which could be combined with other baseline variables for intra-arrest prognostication. For this purpose, the 20-min survival model achieved the highest discriminative performance in predicting survival to hospital discharge.

## Supplementary Information




