# Supplementary material for: End-tidal Carbon Dioxide Trajectory-based Prognostication of Out-of-hospital Cardiac Arrest
Source: West J Emerg Med. 2024 Jun 11;25(4):521–32. doi: 10.5811/westjem.18403 (PMC11254150; doi:10.5811/westjem.18403)
Supplement: Supplementary file 1 [file wjem-25-521-s001.docx]

**Supplemental Table 1.** **Characteristics of patients included in the twenty-min group stratified by survival to hospital discharge**

| Variables | Twenty-min group (n=542) | Survival to hospital discharge (n=25) | Death at hospital discharge (n=517) | *p* value |
| --- | --- | --- | --- | --- |
| Basic demographics |  |  |  |  |
| Age, year | 68.0 (57.0-80.0) | 60.0 (50.8-63.8) | 69.0 (57.0-81.0) | <0.001 |
| Male, n | 354 (65.3) | 21 (84.0) | 333 (64.4) | 0.05 |
| Peri-CPR events |  |  |  |  |
| Transported by EMS, n | 507 (93.5) | 22 (88.0) | 485 (93.8) | 0.25 |
| Arrest at home, n | 296 (54.6) | 6 (24.0) | 290 (56.1) | 0.002 |
| Witness by bystander, n | 193 (35.6) | 17 (68.0) | 176 (34.0) | <0.001 |
| Witness by EMS, n | 28 (5.2) | 0 (0) | 28 (5.4) | 0.23 |
| Witness by bystander or EMS, n | 212 (39.1) | 17 (68.0) | 195 (37.7) | 0.003 |
| Bystander CPR, n | 269 (49.6) | 17 (68.0) | 252 (48.7) | 0.06 |
| Prehospital defibrillation by EMS, n | 117 (21.5) | 16 (64.0) | 101 (19.5) | <0.001 |
| Initial shockable rhythms at ED arrival, n | 37 (6.8) | 11 (44.0) | 26 (5.0) | <0.001 |
| Duration of prehospital CPR performed by EMS, min | 17.0 (12.0-21.0) | 15.0 (12.0-19.0) | 17.0 (16.0-18.0) | 0.38 |
| Procedures during CPR |  |  |  |  |
| SGA use, n | 376 (69.4) | 20 (80.0) | 356 (68.9) | 0.24 |
| Time to SGA use, min | 0 (0-0) (n=376) | 0 (0-0) (n=20) | 0 (0-0) (n=356) | 0.53 |
| ETT use, n | 531 (98.0) | 25 (100) | 506 (97.9) | 0.12 |
| Time to ETT use, min | 3.0 (2.0-5.0) (n=531) | 4.0 (3.0-6.3) (n=25) | 3.0 (2.0-5.0) (n=506) | 0.06 |
| Available measurements of EtCO_2_ levels, times | 8.0 (6.0-9.0) | 7.0 (5.0-8.0) | 8.0 (6.0-9.0) | 0.01 |
| Duration of CPR performed in ED, min | 31.0 (30.0-35.0) | 28.0 (24.3-31.5) | 31.0 (30.0-35.0) | <0.001 |
| Outcome, n |  |  |  |  |
| ROSC | 184 (33.9) | 25 (100) | 159 (30.8) | <0.001 |

Data are presented as median (interquartile range) or counts (proportion). CPR, cardiopulmonary resuscitation; ED, emergency department; EMS, emergency medical service; ETT, endotracheal tube; ROSC, return of spontaneous circulation; SGA, supraglottic airway.

**Supplemental Table 2.** **Characteristics of patients included in the ten-min group stratified by survival to hospital discharge**

| Variables | Ten-min group (n=532) | Survival to hospital discharge (n=34) | Death at hospital discharge (n=498) | *p* value |
| --- | --- | --- | --- | --- |
| Basic demographics |  |  |  |  |
| Age, year | 71.0 (59.5-82.0) | 62.5 (53.0-73.0) | 72.0 (60.0-82.0) | 0.05 |
| Male, n | 346 (65.0) | 27 (79.4) | 319 (64.1) | 0.07 |
| Peri-CPR events |  |  |  |  |
| Transported by EMS, n | 500 (94.0) | 31 (91.2) | 469 (94.2) | 0.48 |
| Arrest at home, n | 308 (57.9) | 13 (38.2) | 295 (59.2) | 0.02 |
| Witness by bystander, n | 192 (36.1) | 22 (64.7) | 170 (34.1) | <0.001 |
| Witness by EMS, n | 26 (4.9) | 1 (2.9) | 25 (5.0) | 0.59 |
| Witness by bystander or EMS, n | 207 (38.9) | 22 (64.7) | 185 (37.1) | 0.001 |
| Bystander CPR, n | 276 (51.9) | 23 (67.6) | 253 (50.8) | 0.06 |
| Prehospital defibrillation by EMS, n | 101 (19.0) | 16 (47.1) | 85 (17.1) | <0.001 |
| Initial shockable rhythms at ED arrival, n | 30 (5.6) | 8 (23.5) | 22 (4.4) | <0.001 |
| Duration of prehospital CPR performed by EMS, min | 17.0 (12.0-21.0) | 16.0 (12.0-19.0) | 18.0 (12.0-21.0) | 0.13 |
| Procedures during CPR |  |  |  |  |
| SGA use, n | 380 (71.4) | 26 (76.5) | 354 (71.7) | 0.50 |
| Time to SGA use, min | 0 (0-0) (n=380) | 0 (0-0) (n=26) | 0 (0-0) (n=354) | 0.47 |
| ETT use, n | 508 (95.5) | 34 (100) | 474 (95.2) | 0.19 |
| Time to ETT use, min | 3.0 (2.0-4.0) (n=508) | 3.5 (2.0-5.0) (n=34) | 3.0 (2.0-4.0) (n=474) | 0.05 |
| Available measurements of EtCO_2_ levels, times | 4.0 (3.0-5.0) | 4.0 (3.0-5.0) | 4.0 (3.0-5.0) | 0.25 |
| Duration of CPR performed in ED, min | 30.0 (18.0-32.0) | 19.5 (13.0-26.0) | 30.0 (19.0-32.0) | <0.001 |
| Outcome, n |  |  |  |  |
| ROSC | 239 (44.9) | 34 (100) | 205 (41.2) | <0.001 |

Data are presented as median (interquartile range) or counts (proportion). CPR, cardiopulmonary resuscitation; ED, emergency department; EMS, emergency medical service; ETT, endotracheal tube; ROSC, return of spontaneous circulation; SGA, supraglottic airway.

**Supplemental Table 3. Prediction models with bootstrapped results.**

| Variables | Odds ratio (95% confidence interval) | *p* value |
| --- | --- | --- |
| *Twenty-min survival model* |  |  |
| Twenty-min EtCO_2_ trajectory | 2.25 (1.04-4.86) | 0.04 |
| Arrest at home | 0.28 (0.09-0.87) | 0.03 |
| Prehospital defibrillation by EMS | 3.42 (1.06-11.07) | 0.04 |
| Initial shockable rhythms at ED arrival | 8.36 (2.45-28.57) | 0.001 |
| *Twenty-min ROSC model* |  |  |
| Twenty-min EtCO_2_ trajectory | 2.46 (1.74-3.47) | <0.001 |
| Arrest at home | 0.54 (0.34-0.87) | 0.01 |
| Witness by bystander or EMS | 1.72 (1.09-2.73) | 0.02 |
| Prehospital defibrillation by EMS | 2.72 (1.58-4.67) | <0.001 |
| Initial shockable rhythms at ED arrival | 4.97 (1.81-13.60) | 0.002 |
| Duration of prehospital CPR performed by EMS | 0.96 (0.93-0.99) | 0.004 |
| *Ten-min survival model* |  |  |
| Ten-min intermediate or high EtCO_2_ trajectory | 2.53 (0.007-0.04) | 0.05 |
| Witness by bystander | 3.00 (1.34-6.70) | 0.007 |
| Initial shockable rhythms at ED arrival | 5.21 (1.70-15.98) | 0.004 |
| *Ten-min ROSC model* |  |  |
| Ten-min intermediate EtCO_2_ trajectory | 3.36 (2.28-4.96) | <0.001 |
| Ten-min high EtCO_2_ trajectory | 6.59 (3.36-12.92) | <0.001 |
| Age between 37 and 69 (year) | 1.49 (1.00-2.23) | 0.05 |
| Witness by bystander or EMS | 1.92 (1.30-2.85) | 0.001 |
| Initial shockable rhythms at ED arrival | 5.29 (1.76-15.94) | 0.003 |
| Duration of prehospital CPR performed by EMS (min) | 0.96 (0.93-0.98) | <0.001 |

CPR: cardiopulmonary resuscitation; ED: emergency department; EMS: emergency medical service; ROSC: return of spontaneous circulation.
